# Neutrophils and Platelets as Key Players in the Pathogenesis of ANCA-Associated Vasculitis and Potential Sources of Disease Activity Biomarkers

**DOI:** 10.3390/diagnostics15151905

**Published:** 2025-07-29

**Authors:** Anna Drynda, Marcin Surmiak, Stanisława Bazan-Socha, Katarzyna Wawrzycka-Adamczyk, Mariusz Korkosz, Jacek Musiał, Krzysztof Wójcik

**Affiliations:** 1 Doctoral School of Medical Sciences and Health Sciences, Jagiellonian University Medical College, 31-530 Cracow, Poland; anna.drynda@doctoral.uj.edu.pl; 2 2nd Department of Internal Medicine, Faculty of Medicine, Jagiellonian University Medical College, 30-688 Cracow, Poland; marcin.surmiak@uj.edu.pl (M.S.); stanislawa.bazan-socha@uj.edu.pl (S.B.-S.); katarzyna.wawrzycka@uj.edu.pl (K.W.-A.); jacek.musial@uj.edu.pl (J.M.); 3 Department of Rheumatology and Immunology, Jagiellonian University Medical College, 30-688 Cracow, Poland; mariusz.korkosz@uj.edu.pl

**Keywords:** ANCA-associated vasculitis (AAV), neutrophils, platelets, myeloperoxidase (MPO), proteinase-3 (PR3), neutrophil extracellular traps (NETs), extracellular vesicles (EVs), biomarkers

## Abstract

Anti-neutrophil cytoplasmic antibodies (ANCA)-associated vasculitis (AAV) is a heterogeneous group of small-vessel vasculitides, characterized by the presence of antibodies binding to myeloperoxidase (MPO) and proteinase-3 (PR3) found in neutrophil granules. Apart from being the target of ANCA, neutrophils actively contribute to the vicious cycle of inflammation and vascular damage in AAV. On the other hand, platelets have recently been recognized as essential for thrombosis and as inflammatory effectors that collaborate with neutrophils, reinforcing the generation of reactive oxygen species (ROS) and the formation of neutrophil extracellular traps (NETs) in those diseases. Neutrophils exhibit morphological and functional heterogeneity in AAV, reflecting the complexity of their contribution to disease pathogenesis. Since long-term immunosuppression may be related to serious infections and malignancies, there is an urgent need for reliable biomarkers of disease activity to optimize the management of AAV. This review summarizes the current understanding of the role of neutrophils and platelets in the pathogenesis of granulomatosis with polyangiitis (GPA) and microscopic polyangiitis (MPA), focusing on their crosstalk, and highlights the potential for identifying novel biomarkers relevant for predicting the disease course and its relapses.

## 1. Introduction

The anti-neutrophil cytoplasmic antibodies (ANCA)-associated vasculitis (AAV) comprises a group of autoimmune diseases characterized by inflammation of small and medium-sized blood vessels, often leading to multi-organ damage. According to the 2012 Revised International Chapel Hill Consensus Conference Nomenclature, AAV encompasses three main subtypes: granulomatosis with polyangiitis (GPA), microscopic polyangiitis (MPA), and eosinophilic granulomatosis with polyangiitis (EGPA) [1], with EGPA being the rarest and most distinct form due to its eosinophilic component. The presence of ANCA is a characteristic feature of AAV, with PR3-ANCA (proteinase-3 ANCA) associated with GPA and MPO-ANCA (myeloperoxidase ANCA) observed in MPA. Unlike GPA and MPA, where ANCA is detected in approximately 90% of cases, only 30–40% of EGPA cases are ANCA-positive, primarily with MPO-ANCA [2,3]. The heterogeneity of AAV presents significant challenges in diagnosis and management, as symptoms and disease severity vary.

Although any tissue can be involved, the respiratory tract and kidneys are most frequently affected, with complications such as pulmonary hemorrhage and rapidly progressive glomerulonephritis (RPGN) posing serious risks, including death [3,4,5]. Additionally, some patients exhibit overlapping features of different AAV subtypes, further complicating classification and prognosis [2,6,7]. Finally, the clinical presentation and disease trajectory may also change, making the long-term prognosis and treatment plans complex and challenging [8].

Given the typical relapse-remission pattern of AAV, remission induction and remission maintenance are fundamental paradigms in management. Remission induction in GPA and MPA depends on the severity of disease presentation. For patients with life- or organ-threatening manifestations, both the 2021 American College of Rheumatology/Vasculitis Foundation (ACR/VF) [9] and 2022 European Alliance of Associations for Rheumatology (EULAR) guidelines [10] recommend a combination of glucocorticoids (GC) and either rituximab (RTX) or cyclophosphamide (CYC). Maintenance of remission relies on intensive and long-term immunosuppression. In GPA and MPA, maintenance treatment with repeated doses of RTX is superior to azathioprine (AZA) and methotrexate (MTX) in reducing relapse rates, while AZA and MTX are recommended when RTX is contraindicated [11,12]. Mycophenolate mofetil (MMF) is considered a third-line treatment [9,10]. However, in some patients, discontinuing GC despite immunosuppressive therapy use may still be unfeasible [10].

Immunosuppressive treatment in AAV patients is associated with various complications, some of which can be severe and potentially fatal, particularly during long-term maintenance therapy. It has been shown that infections and malignancies rank among the top three long-term causes of death in AAV, being surpassed only by cardiovascular diseases, whose risk increases dose-dependently with GC use [13,14,15]. Therefore, it is crucial to balance therapy to reduce the risk of disease relapse and also minimize the side effects and toxicity of immunosuppression. Since no reliable clinical parameters are available, laboratory biomarkers are urgently needed to predict clinical trajectory and disease relapse.

Vasculitides are related to the inflammatory process that damages the walls of small and medium-sized blood vessels, resulting in impaired blood flow and ischemia [16,17]. The exact mechanisms underlying immunologic and cell responses, including preliminary triggers, have not been fully understood; however, the central role of neutrophils in the pathogenesis of AAV is unquestionable [18,19,20,21]. Platelets, on the other hand, have recently been considered as potential contributors to the inflammatory process underlying AAV. Moreover, there is growing evidence of crosstalk between neutrophils and platelets in various conditions [22,23,24], including AAV [25,26,27,28]. Given the relatively novel evidence on platelet involvement in AAV pathogenesis, we aim to review that issue. Furthermore, we recapitulate the known facts on the interplay between neutrophils and platelets in AAV and highlight the impact of this novel research on disease prognosis and optimal therapeutic approach in those patients.

## 2. Materials and Methods

A literature search was performed on PubMed and Embase for studies published in English up until April 2025, using MeSH and Emtree terms related to ANCA-associated vasculitis, neutrophils, and platelets. The titles and abstracts were screened for relevance to the review topic. Articles that did not provide new insights were excluded. Additional relevant articles were identified through citation searching. Due to substantially different pathogenesis and rarity, this review did not include data on EGPA.

## 3. Pathogenesis of ANCA-Associated Vasculitis—An Overview

The pathogenesis of AAV involves a complex interplay between innate and adaptive immunity. While ANCA and neutrophils are central to this process, their pathogenicity is modulated and amplified through interactions with monocytes, macrophages, B and T lymphocytes, the complement and coagulation systems, as well as platelets.

Monocytes and macrophages also contribute to vascular damage and immune activation. Circulating monocytes infiltrate inflamed tissues, differentiate into macrophages, and promote granulomatous inflammation. Monocytes are stimulated by ANCA to produce proinflammatory cytokines, such as TNF and IL-6 [29], and are a major source of tissue factor (TF) that links inflammation to thrombosis [30]. They also play a role in impaired clearance of apoptotic neutrophils, which may perpetuate inflammation and interfere with resolution [31]. Additionally, macrophages may respond to NET-derived molecules [32,33] and participate in antigen presentation and T-cell activation, thus bridging innate and adaptive immunity [34].

B cells are essential for the production of ANCA and also serve as antigen-presenting cells. Abnormal B-cell activation and survival, enhanced by elevated B-cell activating factor (BAFF), are observed in AAV and correlate with disease activity [35]. The clinical efficacy of rituximab confirms their crucial pathogenic role in AAV [36]. T cells, including Th1, Th17, CD8+, and regulatory T-cell subsets, are actively involved in both granulomatous inflammation and humoral activation. Dysregulated T-cell function contributes to ANCA production, tissue infiltration, and loss of tolerance. IL-17-producing Th17 cells are increased in active disease, and T cells specific for complementary PR3 peptides (cPR3) have been detected in patients with PR3-ANCA vasculitis [37,38].

The complement system is a crucial amplifier of inflammation. C5a promotes neutrophil priming and chemotaxis [39]. Crosstalk between complement and coagulation systems facilitates thrombin generation and propagates endothelial injury [40]. The coagulation cascade is closely intertwined with immune activation. AAV patients exhibit increased thrombotic risk, associated with neutrophil- and monocyte-derived tissue factors and thrombin-mediated platelet activation [30,41]. Thrombin not only promotes fibrin formation but also stimulates inflammation through PAR signaling, further connecting thrombosis and vasculitis pathophysiology [26,42].

## 4. Neutrophils

### 4.1. Cytokine-Dependent Regulation of Neutrophil Production Is Abnormally Upregulated in AAV

Neutrophils, or polymorphonuclear (PMN) leukocytes, are the most abundant type of leukocytes, constituting 50–70% of their total population. The average lifespan of neutrophils is relatively short, limited by programmed cell death, ranging from 7 to 9 h [43] to over 5 days [44]. Therefore, to maintain their count, about 1–2 × 10^11^ neutrophils are produced daily in the bone marrow via neutropoiesis [45], a process dependent on granulocyte colony-stimulating factor (G-CSF) activity [46]. After maturation, neutrophils are retained in bone marrow for the next 4–6 days, forming a reserve pool, ready to be deployed in case of an infection, inflammation, or trauma [47]. Neutrophil retention and release are regulated by chemokine signaling, the expression of which is controlled also by G-CSF [48]. In MPA, high initial levels of circulating G-CSF were associated with the severity of central nervous system involvement and disease activity, according to the Birmingham Vasculitis Activity Scale (BVAS) [49]. After fulfilling a biological function, neutrophils undergo apoptosis in the bone marrow, spleen, or liver. Then, they are cleared by macrophages through efferocytosis [31], which modulates the neutropoiesis by a negative feedback loop. Macrophages release large amounts of interleukin (IL)-23, triggering IL-17 production by T, NK, and NKT cells, and further increasing G-CSF production. Interestingly, phagocytosis of neutrophils reduces IL-23 production by macrophages, leading to diminished neutropoiesis and cell release to the peripheral blood. On the other hand, when inflammation is amplified, the same cytokine axis intensifies neutrophil proliferation and maturation [50,51]. Patients with AAV exhibit a markedly elevated circulating neutrophil count [52]. In AAV, according to the study by Nogueira et al. [53], serum IL-17A levels were significantly higher in the acute phase of GPA than in healthy controls. Moreover, in the same study, patients with elevated IL-23 had more active disease and higher ANCA titers. Additionally, a study by Huang et al. documented a positive correlation between the neutrophil-to-lymphocyte ratio (NLR) and C-reactive protein (CRP) and an inverse with C3 level in those with MPO-ANCA [54]. Interestingly, a threshold NLR of at least 5.9 was related to increased disease severity and risk of relapse in follow-up [55]. Furthermore, an elevated delta neutrophil index (DNI) of at least 0.65, reflecting the proportion of immature granulocytes within the total neutrophil count, was associated with more severe disease at diagnosis; thus, it could likely serve as a predictor of relapse in GPA or MPA [56].

### 4.2. Neutrophil-Endothelium Interactions Mediate Adhesion, Migration and Inflammatory Amplification in AAV

**Figure 1:**
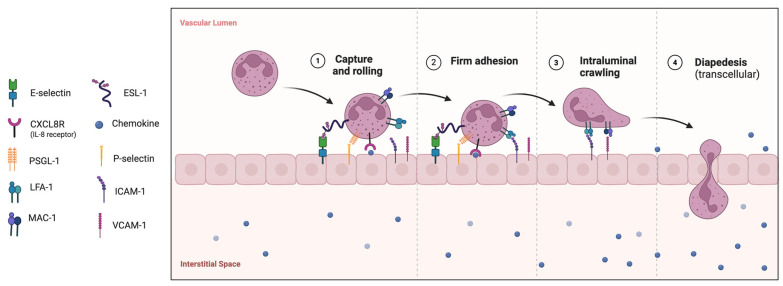
Neutrophil adhesion cascade preceding extravasation. This process involves capture, rolling, activation, firm adhesion, and intraluminal crawling. It depends on interactions of neutrophil glycoproteins with endothelial E- and P-selectins, whose expression is upregulated in inflammatory loci. Firstly, E- and P-selectin capture circulating neutrophils to the surface of endothelium by interacting with E-selectin ligand-1 (ESL-1) and P-selectin glycoprotein ligand 1 (PSGL1), respectively. The interaction of β_2_ integrins, namely lymphocyte function-associated antigen-1 (LFA-1, α_L_β_2_, CD11a/CD18) and macrophage-1 antigen (Mac-1, α_M_β_2,_ CD11b/CD18) with ligands such as vascular cell adhesion molecule (VCAM-1), intercellular adhesion molecule-1 (ICAM-1, CD54), and ICAM-2 on endothelial cells, ensures firm adhesion of neutrophils.

As vascular wall damage in small vessels plays a key role in AAV, interactions between neutrophils and the endothelium are crucial to its pathogenesis. Under physiological conditions, neutrophils do not adhere to resting endothelium. Upon exposure to inflammatory stimuli, neutrophils migrate towards the inflammation site, where they undergo adhesion cascade and extravasation (Figure 1) [57,58,59].

The expression of adhesion molecules, along with the deposition of plasma fibronectin on leukocytes, is enhanced by inflammation and endothelial damage. Both ANCA and tumor necrosis factor (TNF) increase β_2_ integrin expression on neutrophils, further promoting their attachment and neutrophil aggregation, enhancing trapping of cell aggregates in capillaries [60]. Adhesion of neutrophils is followed by crawling along the surface of the endothelium, following the gradient of chemoattractants, adhesion receptors, and endothelial stiffness. Finally, neutrophils travel through the vascular wall via paracellular or, to a lesser extent, transcellular routes in the process of diapedesis, facilitated by the interaction of β_2_ integrins, ICAM-1, platelet endothelial cell adhesion molecule 1 (PECAM-1), and junctional adhesion molecules [61]. In this context, the biological role of CD177, a novel IgG Fc receptor found on the neutrophil surface, should also be considered. It has been demonstrated that CD177 ligation causes the migratory arrest of neutrophils, likely triggered by β_2_ integrins as signaling partners, including their higher expression and affinity, reduced internalization, and extracellular signal-regulated kinases (ERK)-driven suppression of chemoattractants stimuli, potentially leading to endothelial injury [62]. Next, studies on murine models and in vitro have shown that some adhesive molecules can increase neutrophil extracellular trap (NETs) formation, a process essential to AAV pathogenesis and tremendously enhancing local inflammatory response. For instance, Mac-1 augments lipopolysaccharides (LPSs)-related NET formation (NETosis), P-selectin after ligation to PSGL-1, while the β_2_ integrin subfamily when triggered by β-glucan [60].

Semaphorin A4D (SEMA4D) is a glycoprotein engaged in neutrophil-endothelium interactions, considered a potential biomarker of disease activity. In typical physiological conditions, the membrane complex of CD100/SEMA4D serves as an inhibitory receptor on neutrophils, where it interacts with plexin-B2 (PLXNB2) expressed by endothelial cells. This interaction shields the endothelium from damage, mainly by suppressing neutrophil-driven immune responses by reducing the production of reactive oxygen species (ROS) and NETosis [63]. A disintegrin and metalloproteinase domain-containing protein 17 (ADAM17) is a protease responsible for cleavage membrane-bound proteins, including SEMA4D, converting it to a soluble form [63,64]. A study by Bertram et al. [64] has reported a higher circulating ADAM17 concentration in patients with active PR3-AAV with kidney involvement compared to those in remission or with nonvascular kidney diseases. Similarly, markedly elevated serum SEMA4D levels have been demonstrated in patients with AAV, depicting a positive association with the BVAS score, and they were higher than in other autoimmune diseases, such as rheumatoid arthritis or systemic lupus erythematosus (SLE) [63]. Furthermore, Wang et al. [65] demonstrated that neutrophils from AAV patients exhibited a notable reduction in cell-surface SEMA4D compared to healthy controls. These findings suggest increased proteolytic shedding of SEMA4D in AAV, likely mediated by ADAM17. Since membrane-bound SEMA4D contributes to endothelial protection by regulating neutrophil activation, its loss from the cell surface, resulting in elevated levels of soluble SEMA4D, may impair this regulatory mechanism and promote neutrophil-mediated vascular injury.

### 4.3. Neutrophil Granule Proteins, Reactive Oxygen Species Production and Extracellular Vesicles Shape the Inflammatory Environment in AAV

Neutrophils possess an armamentarium of mechanisms aimed at pathogen detection and destruction. Neutralization of ingested particles and pathogens relies on two main cytotoxic mechanisms—ROS generation in the oxidative burst, catalyzed by nicotinamide adenine dinucleotide phosphate (NADPH) oxidase [66], and activity of antibacterial proteins, originating from neutrophil granules [67].

Depending on the stage of neutrophil differentiation, the granules can be divided into four main types—azurophil, specific, gelatinase, and secretory [68]. Degranulation through exocytosis also serves as a targeted, defensive mechanism and further enhances inflammatory response, with secretory granules being the most available for exocytosis [69]. Azurophil granules, however, are particularly relevant in the context of autoimmune vasculitis. Among various potent antimicrobial proteins—such as defensins, cathelicidins, cathepsin B, and lysozyme—they also include MPO and PR3 [70,71,72]. This phenomenon is mainly related to epigenetic dysregulation [73]. Furthermore, expression of these proteins on the neutrophil surface is augmented upon stimulation by complement component 5a (C5a) and cytokines released after ANCA binding [74].

**Figure 2:**
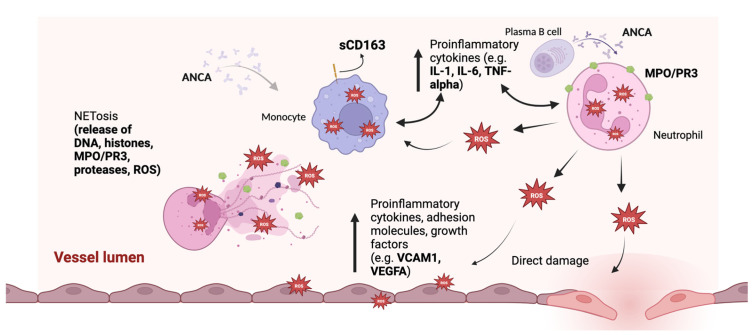
Reactive oxygen species (ROS) play a critical role in AAV. In addition to direct endothelial damage and perpetuating the inflammation via cytokine release, ROS generation is crucial for initiating NETosis. Monocytes and macrophages stimulated by ANCA, ROS, and inflammatory mediatiors release soluble CD163, which urine levels are a relatively novel biomarker of kidney vasculitis flare [83].

ROS damage cellular components by causing double-stranded DNA breaks [75], inducing lipid peroxidation that compromises membrane integrity [76], and oxidizing proteins, leading to their misfolding and loss of function [77]. Hydroperoxides of amino acids and their residues are generated during oxidative modification driven by ROS, propagating oxidative damage further within proteins [78]. In addition to direct endothelium damage, oxidative burst might play a role in the stimulation of NETosis [79] (Figure 2), as its inhibition decreases NET formation [80]. A study by Hilhorst et al. [81] suggested that kidney damage in AAV depends on an imbalance between ROS and antioxidant defenses. Oxidative burst and hypochlorous acid (HOCl) production in neutrophils due to MPO activation at diagnosis have been linked to the presence of active cellular crescents in MPA, whereas higher serum thiol concentrations correlated with fewer cellular crescents and reduced interstitial fibrosis. According to the same study, MPA patients display higher oxidative stress, characterized by increased serum HOCl and advanced oxidation protein products (AOPP) concentration compared to healthy individuals. Interestingly, Sun et al. [82] reported a decrease in AAV-associated organ damage and MPO deposition in the kidneys and lungs of rat models treated with astaxanthin, a potent antioxidant. Therefore, the level of systemic oxidative stress response could reflect disease activity in AAV, making it a promising direction for further research.

Beyond degranulation and ROS production, neutrophils also mediate inflammation via extracellular vesicles (EVs). EVs are an umbrella term for phospholipid bilayer-enclosed vesicles released by eukaryotic cells. Depending on their size, EVs can be divided into exosomes (50–150 nm) and microparticles (100–1000 nm) [84]. EVs regulate cell-to-cell communication, with different stimuli influencing the composition of their cargo, including proteins, nucleic acids, and lipids [85]. EVs cargo has been profiled in numerous studies investigating potential biomarkers in various conditions, such as cancer [86,87], neurological disorders [88], autoimmune diseases [89,90], and thrombosis [91]. Neutrophil-derived EVs were shown to carry ANCA-antigens, proinflammatory miRNA, and oxylipins. Interestingly, studies report a positive correlation between the BVAS and the levels of C3a, C5a, pentraxin 3 (PTX3) [92], and high mobility group box 1 (HMGB1) expressed on MPO-positive microparticles [93,94]. A recent study by our team [95] has reported that EVs from GPA patients, likely originating from neutrophils, encompass higher leukotriene B_4_ (LTB_4_) and 5-oxo-eicosatetraenoic acid (5-oxo-ETE) content compared to EVs from healthy controls. This study also shows that primed neutrophils stimulated with LTB_4_ or 5-oxo-ETE exhibit a concentration-dependent increase in ROS production and dsDNA release. Another study of ours [96] shows that EVs released by IgG anti-PR3-activated neutrophils induce human umbilical vein endothelial cells (HUVECs) to produce proinflammatory cytokines via their miRNA content (most notably miR-223-3p and miR-142-3p). Furthermore, the expression of EVs-derived miR-223-3p and miR-664a-3p has been elevated in active GPA and correlated positively with BVAS, but also with circulating DNA-MPO complexes, suggesting a link to NETosis [97]. In a study by Glémain et al. [98], miR-142-3p and miR-451 from neutrophil-derived EVs have been implicated in promoting endothelial inflammation and damage. Furthermore, increased expression of miR-223, miR-142-3p, and miR-451 has been observed in peripheral blood of those with kidney involvement, suggesting their role in AAV progression.

### 4.4. Phenotypic Diversity of Neutrophils, Including CD177^+^ and Low-Density Subsets, Is Associated with Disease Activity in AAV

In the past, neutrophils were thought to form a homogenous population of cells possessing uniform properties. However, this view has been challenged by findings enabled by the advent of high-resolution technologies [70]. Despite their short lifespan, neutrophils change in the process of aging. In addition to downregulating CXCR2 and L-selectin, it manifests by upregulating CXCR4, CD11b, and CD49 [99], accompanied by increased capacity to form NETs. These processes can be linked to the activation of pathogen-recognition receptors by pathogen associated molecular patterns (PAMPs) [100] and depend on changes in cortisol level and expression of circadian-clock genes, remaining in relation to circadian rhythm [101]. The abovementioned alterations promote neutrophils’ homing and infiltrating tissues, where they act as sentinels. In the lungs, for instance, neutrophils adhere to the vascular lumen and reside in the interstitial spaces, retained by a mechanism that depends on CXCR4, making them a possible destination for aged neutrophils [102]. Evidence indicates that neutrophils might display differences in phenotype also across tissue [103,104]. Moreover, neutrophils display substantial diversity in morphology and function in pathological contexts of inflammation, infection, and cancer [105]. For instance, the proportion of circulating neutrophils expressing CD177 remains stable within an individual, regardless of age, gender, or activation state. However, their percentage can increase during pregnancy [106], GM-CSF therapy, or in patients with polycythemia vera [107]. Studies have revealed that variability in CD177 expression affects the surface display of PR3 [108]. Only CD177(+) neutrophils express PR3, and an increased proportion of these cells is linked to a higher risk of developing AAV [109] and a higher risk of relapse in GPA [110]. Stimulation of CD177 triggers degranulation and ROS production, especially in neutrophils expressing high levels of CD177 and PR3 [111]. Interestingly, that effect is attenuated by blocking the complement receptor integrin Mac-1 [112]. On the other hand, neutrophils of MPO-ANCA-positive individuals exhibit higher expression of LFA-1 compared to PR3-ANCA-positive ones, with this integrin expression associated with systemic and pulmonary components of the BVAS [113]. Furthermore, in AAV, neutrophils coexpressing CD177, LFA-1, Mac-1, CD80, CD86, CD100/SEMA4D, class II major histocompatibility complex (MHC II), TNF receptor (TNFR)1, and TNFR2 are detected in higher percentages, also in positive correlation with the BVAS [63,113,114,115].

Among neutrophil subsets described in a pathological context, particular attention is given to low-density neutrophils (LDNs), first identified in individuals with SLE [116]. LDNs can be separated from other granulocytes using a discontinuous density gradient and exhibit a density comparable to peripheral blood mononuclear cells (PBMCs). They have been observed in various conditions, including cancer [117], infections [118], and autoimmune disorders, such as rheumatoid arthritis [119] and psoriasis [120]. Studies based on autoimmune conditions suggest that LDGs are characterized by more pronounced proinflammatory properties [121,122], a higher tendency to undergo NETosis [123,124], and better promotion of Th17 cell differentiation compared to other subsets of neutrophils [125]. Interestingly, LDGs consisting of both mature CD10(+) and immature CD10() cells have been identified in AAV [126]. The CD10(−) subset displays downregulated CD16 and CD88/C5aR expression, an immature nuclear structure, and diminished responsiveness to stimulation by MPO-ANCA, which undermines their relevance in AAV pathogenesis [126]. On the other hand, LDGs co-expressing CD10, CD16, and CD88/C5aR can be induced by ANCA stimulation and likely play a role in activating the alternative complement pathway [127]. LDGs have been reported to correlate with disease activity in AAV [128]. However, it is important to note that LDNs encompass a broad spectrum of neutrophil phenotypes and have been observed in lower fraction also in healthy individuals [129]. The actual distinctiveness and relevance of neutrophil phenotypes have been questioned and attributed to the limitations of cell isolation methodology, making it one of the most controversial current aspects of neutrophil research [130,131].

### 4.5. Neutrophils Exhibit a Decreased Rate of Spontaneous Apoptosis and Enhanced NETosis in AAV

After completing their function, senescent neutrophils are removed from the inflamed tissue to avoid unnecessary perpetuation of the inflammation [70]. Besides homing to undergo apoptosis and efferocytosis, they can migrate in reverse or perish via different death processes, including pyroptosis, ferroptosis, necroptosis, necrosis, and NETosis [132]. In AAV, neutrophils show a decreased rate of spontaneous apoptosis in vitro, resulting in a prolonged lifespan, which suggests a tendency to accumulate at the inflammation site in vivo [133]. Delayed apoptosis observed in neutrophils of GPA patients involves both the intrinsic and extrinsic pathways and is likely driven by elevated levels of circulating factors such as GM-CSF, TNF, and soluble Fas cell surface death receptor (sFas) [134]. On the other hand, the opposite situation occurs in the case of NETosis in AAV. NETosis is a distinct form of cell death—as opposed to apoptosis, it involves the disintegration of neutrophil cell membrane paired with the release of NETs, which consist of granule contents combined with double-stranded DNA. This process is initiated by ROS generated through NADPH oxidase, which stimulates MPO to activate neutrophil elastase (NE) and promotes its translocation from azurophilic granules into the nucleus. There, NE cleaves histones and facilitates chromatin decondensation, aided by MPO, which acts synergistically. To reach the nucleus, NE must first degrade cytoplasmic F-actin. Notably, during NETosis, plasma membrane permeabilization occurs in a programmed manner, not due to physical disruption by expanding chromatin [135]. Upon release, chromatin immobilizes pathogens, and granule-derived proteins exert their antimicrobial effect, making this process a type of cell death and an effector neutrophil mechanism [136]. Moreover, it has been observed that, as in the case of unregulated necrosis, NETosis can drive a proinflammatory response instead of mitigating it, contributing to organ injury [137]. In AAV, neutrophils display enhanced NETosis compared to healthy individuals [138,139]. Although NET formation in AAV is often driven by ANCA, Kraaij et al. [139] suggest it can occur independently of them. Even when IgG and IgA were depleted from AAV patient serum, NETosis still occurred in vitro, indicating the presence of additional humoral factors contributing to neutrophil activation and disease progression. MPO and PR3 are integral components of NETs, so their exposure may further drive ANCA production. In AAV, NETs enriched with PR3 and MPO have been detected in kidney tissues during active disease [140,141]. Interestingly, while both MPO and PR3 can form complexes with DNA, a study by our group [138] shows that MPO-DNA complexes are consistently present in all GPA patients, whereas PR3-DNA complexes can be detected only in some. Considering this, PR3-DNA complexes are of limited utility as NET formation markers in AAV, while MPO-DNA complexes appear to serve as a universal circulating marker of NET formation. In the same study, patients with active GPA exhibited elevated circulating levels of serine proteases, DNA-histone complexes, and MPO-DNA complexes. However, DNA-histone complexes were similarly elevated in GPA patients in remission and did not correlate with the BVAS score, further supporting the role of MPO-DNA complexes as a more reliable marker of NETosis. Additionally, increased levels of free circulating mitochondrial and genomic DNA have been identified as serum markers of GPA. Elevated circulating nucleosome levels, previously observed in AAV, further highlight the role of NETosis in disease pathology [142]. Moreover, fibrous DNA deposits containing PR3 and MPO can activate plasmacytoid dendritic cells (pDCs) and autoreactive B cells through a Toll-like receptor 9 (TLR-9)–dependent mechanism [143], potentially amplifying the autoimmune response. Interestingly, NET formation is not inextricably associated with cell death, as it can also occur via vital NETosis, contributing to the persistent, pathological immune response [144].

### 4.6. Neutrophil Effector Functions as an Emerging Therapeutic Target in AAV

In addition to their diagnostic and prognostic relevance, neutrophils and their proteases have gained attention as potential therapeutic targets in AAV. Rather than broadly suppressing immune responses, novel approaches aim to selectively inhibit neutrophil effector functions responsible for vascular injury. Preventing NETosis, for instance by PAD4 inhibitors, which prevent chromatin decondensation and NET release [145], seems particularly potential, but has not been evaluated in AAV yet. Similarly, inhibitors of neutrophil serine proteases [146,147] appear to be a compelling research direction in the search for agents to attenuate the inflammatory process in AAV. Although such inhibitors have not yet been evaluated in AAV, a study by Jerke et al. [148] on neutrophils derived from patients with Papillon-Lefèvre syndrome, who have a genetic deficiency in cathepsin C and consequently reduced protease activity, showed impaired neutrophil activation, lower surface expression of PR3, and significantly less endothelial damage. The same study further demonstrated that in human stem cell-derived neutrophils, where a specific cathepsin C inhibitor reduced levels of neutrophil serine proteases, membrane PR3 expression, PR3-ANCA-induced activation, and the transfer of active proteases to glomerular microvascular endothelial cells, which are the primary target in ANCA-induced necrotizing crescentic glomerulonephritis. Small-molecule inhibitors of spleen tyrosine kinase (Syk), central to ANCA-induced neutrophil activation via Fcγ receptors also bear a potential to become a new therapeutic target [149,150]. Notably, avacopan, an oral C5aR1 antagonist that has become a therapeutic option in AAV, prevents neutrophil priming and recruitment [151,152].

## 5. Platelets

Platelets are small, anucleate cells circulating for about 7–10 days in humans before being cleared in the spleen and liver. Platelet production, or thrombopoiesis, takes place primarily in the bone marrow. This process begins with hematopoietic stem cells (HSCs) differentiating into large, polyploid megakaryocytes, which then extend long, cytoplasmic protrusions known as proplatelets, ultimately demarcating their tips and releasing platelets into circulation [153]. Approximately 10^11^ platelets are produced daily [154]. While the roles of platelets in hemostasis and thrombosis have been extensively studied, their contributions to the immune response, particularly in autoimmune diseases, have only recently been recognized.

Although platelet counts are higher in patients with GPA compared to healthy individuals, no significant difference has been observed between those in remission and those with active disease [138,155]. Proposed mechanisms of count increase comprise interactions between platelet-derived sCD40L and matrix metalloproteinase-9 (MMP-9) [155] and between platelet-derived CCL5 and megakaryocytes [156]. A recent study by Lee et al. [157] has proposed a novel predictor based on platelet, neutrophil, monocyte, and lymphocyte counts—pan-immune-inflammation value (PIIV = neutrophil count (×1000/m^3^) × monocyte count (×1000/m^3^) × platelet count (×1000/mm^3^)/lymphocyte count (×1000/m^3^)). A high PIIV (cut-off point of at least 1011.3) at the onset of AAV has been postulated as an independent risk factor for all-cause mortality. Additionally, patients with GPA and MPA—especially those with kidney and pulmonary involvement—exhibit significantly elevated peripheral blood platelet count and platelet-to-lymphocyte ratio (PLR). PLR of 272.0 or greater has been linked to more severe disease activity [158,159]. On the other hand, in a study by Jin et al., lower platelet counts at presentation (cut-off point of 264.5 × 109/L) are suggested to be a predictor of worse kidney function, including end-stage kidney disease (ESKD), and death [160]. This observation is supported by a study by Sánchez Álamo et al., where lower platelet count was a predictor of mortality in AAV; however, the cut off value was within normal limits as well [161].

Platelets possess granules of three types: (1) alpha granules containing chemokines (CXCL7, CXCL4/PF4, CXL1/GROα, CXCL5, CCL5/RANTES, CCL3/MIP1α) [162], coagulation factors, PDGF receptors, TGF-β, P-selectin, fibrinogen, von Willebrand factor (vWF), and fibronectin; (2) dense granules storing calcium, magnesium, nucleotides (ADP, ATP), serotonin, and histamine; and (3) lysosomal granules, including glycohydrolases and proteases such as cathepsin, acid phosphatase, collagenase, and elastase. Dense bodies play a role in vasoconstriction and cytokine production primarily due to their high serotonin content [163,164]. In addition, platelets participate in immune responses by producing and releasing proinflammatory cytokines, most notably IL-1β [165]. They interact with specific receptors on leukocytes, such as monocytes, leading to cell activation. Platelets also express a diverse range of immune receptors, including chemokine receptors [166], Fc receptors [167], TLRs [24,168], and complement receptors [169,170,171]. The complement system plays a vital role in the pathogenesis of AAV [172]. Activation of the complement pathway leads to deposition of membrane attack complex (MAC) on the platelet surface and generation of C3a and C5a, further stimulating platelets [26].

In response to vascular damage, platelets undergo immediate changes in shape and function to participate in hemostasis or contribute to thrombosis in an unfavorable scenario. However, as platelets form heterogeneous subpopulations, chronic diseases involve shifts in their composition, altering platelet phenotypes [173]. Changes in platelet phenotypes can be driven by interactions between fully formed platelets and circulating inflammatory molecules or cells and by modulating platelet production by alterations in the megakaryocyte microenvironment. Such observations have been made in several disorders characterized by prolonged systemic inflammation, such as SLE [174], systemic sclerosis [175], and cancer [176]. For instance, chronic inflammatory states are characterized by increased platelet receptor expression and activity and the loss of surface receptors through cleavage and shedding [173]. However, as this is a relatively novel topic, it remains understudied and highlights a new direction for research in AAV monitoring.

## 6. Neutrophil-Platelet Crosstalk

Recent studies have shown that the progression of AAV involves interactions between activated platelets and neutrophils, as well as the interplay between the coagulation and complement systems

A study by Miao et al. [27] has investigated in AAV platelet-derived microparticles (PMP) containing proinflammatory cytokines, adhesion molecules, and growth factors, and has shown a positive correlation between PMP levels and erythrocyte sedimentation rate (ESR), CRP concentrations, and the BVAS score, particularly in relation to kidney involvement. The PMP level was also positively associated with both platelet and neutrophil counts and the percentage of neutrophils among leukocytes during the active phase of AAV. Upon exposure to cytokines such as TNF, but also C5a, and ANCA, neutrophils undergo an oxidative burst and degranulate, releasing microparticles containing tissue factors, which enhance NET formation. In turn, NETs not only enhance platelet adhesion [177] and aggregation [135], but also contribute to the activation of the coagulation cascade [178].

**Figure 3:**
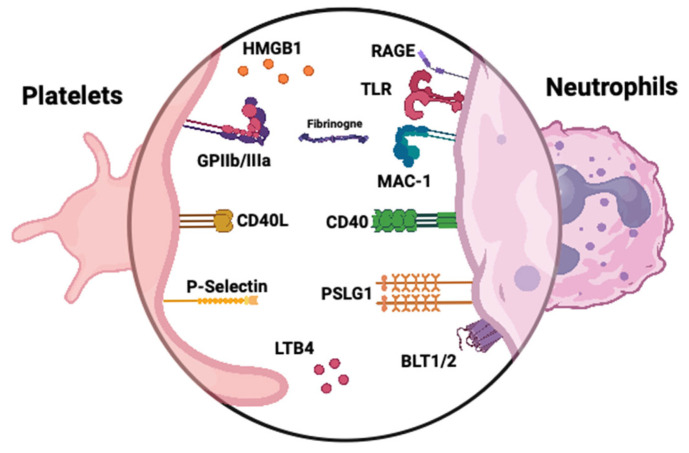
Platelet-neutrophil surface interactions in AAV. Upon activation, platelets express adhesion molecules and release inflammatory mediators that engage corresponding receptors on neutrophils. Notable interactions include P-selectin binding to PSGL-1 and platelet-derived HMGB1 engaging RAGE and TLRs on neutrophils, amplifying the inflammatory response through signaling pathways such as NF-κB and MAPK. Additionally, neutrophil MAC-1 interacts with platelet GPIIb/IIIa, facilitating firm adhesion. Leukotriene B4 (LTB4) and its receptors BLT1/2 contribute to neutrophil recruitment and activation.

Both tissue factor and NETs components initiate the coagulation cascade, ultimately generating thrombin [22]. Thrombin then activates platelets through protease-activated receptors (PARs), leading to platelet degranulation and the release of P-selectin. Platelet receptors facilitate interactions with neutrophils, promoting ROS generation and further NET formation (Figure 3) [25,28]. These interactions damage the endothelium and trigger vWF release, which mediates platelet adhesion and aggregation, exacerbating vascular injury. Meanwhile, the activated neutrophils and platelets, through their membranes, microparticles, and NETs, stimulate the alternative complement pathway, increasing C5a levels. This positive feedback loop amplifies thrombin production and platelet/endothelial activation, perpetuating the inflammatory processes [179,180]. Activated platelets with bound fibrinogen can stimulate neutrophils, triggering an oxidative burst and facilitating neutrophil extravasation. Elevated plasma levels of platelet-derived soluble CD40L and CD62P/P-selectin complex correlate with disease activity in GPA [181]. The interaction between soluble CD40L and CD40 facilitates communication between platelets and endothelial cells, triggering the production of proinflammatory cytokines, such as IL-1β, IL-2, and TNF. This signaling also upregulates the expression of endothelial adhesion molecules, including ICAM-1, VCAM-1, and P-selectin, promoting the recruitment of leukocytes to the site of vascular damage. The sCD40L increases endothelial tissue factor expression and contributes to endothelial dysfunction by stimulating neutrophil ROS production. sCD40L can directly activate neutrophils, trigger ROS release, and upregulate Mac-1 [182]. It has been suggested that soluble P-selectin can serve as a biomarker of platelet activation [183]. The interaction of neutrophils and platelets mediated by P-selectin and PSGL enables their aggregation [184]. Patients with active GPA have more platelet-neutrophil aggregates and higher CD11b expression on neutrophils compared to healthy individuals. The percentage of platelet-neutrophil aggregates among neutrophils increases with BVAS [138]. The release of P-selectin from platelets stimulates NET formation; moreover, blocking its interaction with PSGL-1 substantially inhibits platelet-mediated NETosis [185]. In addition, according to a study by Matsumoto et al., platelets activated through TLR9 signaling release CXCL4, which in turn promotes the formation of NETs [28]. Interestingly, the TLR9–CXCL4 axis is upregulated in AAV. Platelets promote the formation of NETs also via activating neutrophils by TLR4 [168]. HMGB1 is a protein derived from platelets, capable of activating TLR4-dependant NETosis [186]. Higher serum levels of HMGB1 have also been linked to AAV with kidney involvement [187].

Finally, the abovementioned CD177 has been shown not only to recognize β_2_ integrins but also to bind to PR3 and PECAM-1, an immunoglobulin family protein found on endothelial cells, platelets, and neutrophils. However, evidence does not confirm its role in binding platelets to neutrophils or enhancing their interactions [62,188]. Further studies are needed to clarify that issue.

## 7. Biomarker Candidates in AAV Related to Neutrophils and Platelets

A summary of candidate biomarkers discussed in this study was presented in Table 1.

Although multiple studies have identified potential biomarker candidates in AAV, further research is necessary to confirm their clinical relevance and facilitate implementation, as most have not progressed beyond the discovery phase. Key challenges include standardizing assay methodologies, defining cut-offs, and verifying their added value in larger cohorts. International collaborative efforts, along with the development of integrative biomarker panels that combine multiple candidates, could accelerate the translational process.

## 8. Conclusions

Growing evidence shows that all essential neutrophil functions can be altered in AAV and are actively involved in disease pathogenesis. Furthermore, platelets play a crucial role in neutrophil activation and NET formation, ultimately leading to vascular damage. Potential predictors of AAV prognosis and biomarkers of disease activity related to neutrophils and platelets include indices calculated from blood counts (e.g., PLR), serum cytokine levels, surface antigen expression, and NET-associated components. However, further research is necessary to confirm their clinical relevance and facilitate implementation, especially as panels of multiple biomarkers. Neutrophil and platelet phenotypes represent a relatively novel and understudied area; therefore, changes in their composition and activation status may not only offer insights into AAV pathogenesis but also hold potential for disease monitoring.

## Figures and Tables

**Table 1:** An overview of neutrophil- and platelet-related biomarker candidates in AAV.

Biomarker	Source	Detection Method	Mechanism	Clinical Correlation and Relevance
ANCA	Autoantibodies	ELISA, IIF	Directly activate neutrophils via PR3/MPO binding	Established diagnostic and classification marker; titers may correlate with relapse [189].
MPO-DNA complexes	NETs	ELISA	Marker of NETosis, MPO bound to extracellular DNA	Correlate with disease activity and organ involvement [138].
Pentraxin-3 (PTX3)	NETs, activated neutrophils	ELISA	Acute-phase protein associated with NETs	Elevated in active disease; correlates with BVAS [92].
High Mobility Group Box 1 (HMGB1)	Neutrophils, platelets	ELISA	DAMP/alarmin promoting inflammation and NETosis	Increased in active AAV with kidney involvement [187].
Neutrophil-to-lymphocyte ratio (NLR)	Peripheral blood	Hematologic index	Indicator of systemic inflammation and neutrophil burden	Correlates with disease severity and relapse (cut-off ≥ 5.9) [55,190].
Delta Neutrophil Index (DNI)	Peripheral blood	Automated hematology analyzer	Reflects immature granulocyte population	Associated with relapse and severity (cut-off ≥ 0.65) [56].
CD177^+^PR3^+^ neutrophils	Neutrophils	Flow cytometry	Subset expressing surface PR3; more responsive to ANCA	Higher percentage in relapse-prone patients [110].
Low-density neutrophils (LDNs)	Neutrophils	Centrifugation in density gradient and flow cytometry	More pronounced proinflammatory properties and tendency to undergo NETosis	Correlates with BVAS [128], although it is unclear if LDNs form a truly distinct subset.
miR-223, miR-142-3p, miR-664a-3p	Neutrophil-derived EVs	RT-qPCR, miRNA sequencing	Proinflammatory miRNAs from neutrophile-derived vesicles	Correlate with BVAS and NETs [97].
P-selectin/sCD40L	Platelets	ELISA	Markers of platelet activation and endothelial crosstalk	Elevated in active GPA and correlate with NETs [180,184].
Platelet-derived microparticles (PMPs)	Platelets	Flow cytometry, ELISA	Include TF, cytokines; promote NETs, coagulation	Correlate with ESR, CRP, BVAS, kidney involvement [27].
SEMA4D (soluble)	Neutrophils	ELISA	Cleaved from surface; loss facilitates NETosis	Elevated in active PR3-AAV [63].
Pan-Immune Inflammation Value (PIIV)	Peripheral blood	Hematologic index	Composite marker of neutrophil/monocyte/platelet burden	Predicts mortality risk (cut-off ≥ 1011.3) [158].
